# 

**Published:** 1871-08

**Authors:** 


### CONTENTS.

COMMUNICATIONS.

Indications for Extraction of the Teeth ..... 325
Dental Jottings ..... 333
Necrosis of the Lower Jaws ..... 335
Dental Ethics ..... 341
Dentistry in Brazil, South America ..... 346
A New Experience ..... 350
Atrophy of the Teeth ..... 352
Oblivious ..... 360

PROCEEDINGS OF SOCIETIES.

California State Dental Association ..... 361

EDITORIAL.

When Philosophers Differ ..... 368

